# Bio-Logic Builder: A Non-Technical Tool for Building Dynamical, Qualitative Models

**DOI:** 10.1371/journal.pone.0046417

**Published:** 2012-10-17

**Authors:** Tomáš Helikar, Bryan Kowal, Alex Madrahimov, Manish Shrestha, Jay Pedersen, Kahani Limbu, Ishwor Thapa, Thaine Rowley, Rahul Satalkar, Naomi Kochi, John Konvalina, Jim A. Rogers

**Affiliations:** 1 Department of Mathematics, University of Nebraska at Omaha, Omaha, Nebraska, United States of America; 2 College of Information Science and Technology, University of Nebraska at Omaha, Omaha, Nebraska, United States of America; 3 Department of Biology, University of Nebraska at Omaha, Omaha, Nebraska, United States of America; 4 Department of Genetics, Cell Biology and Anatomy, University of Nebraska Medical Center, Omaha, Nebraska, United States of America; CRS4, Italy

## Abstract

Computational modeling of biological processes is a promising tool in biomedical research. While a large part of its potential lies in the ability to integrate it with laboratory research, modeling currently generally requires a high degree of training in mathematics and/or computer science. To help address this issue, we have developed a web-based tool, Bio-Logic Builder, that enables laboratory scientists to define mathematical representations (based on a discrete formalism) of biological regulatory mechanisms in a modular and non-technical fashion. As part of the user interface, generalized “bio-logic” modules have been defined to provide users with the building blocks for many biological processes. To build/modify computational models, experimentalists provide purely qualitative information about a particular regulatory mechanisms as is generally found in the laboratory. The Bio-Logic Builder subsequently converts the provided information into a mathematical representation described with Boolean expressions/rules. We used this tool to build a number of dynamical models, including a 130-protein large-scale model of signal transduction with over 800 interactions, influenza A replication cycle with 127 species and 200+ interactions, and mammalian and budding yeast cell cycles. We also show that any and all qualitative regulatory mechanisms can be built using this tool.

## Introduction

With the goal of understanding the complexities of various biological processes, computational modeling is an important part of Systems Biology. However, despite the excitement around computational systems biology and its potential, it has been difficult to fully utilize modeling as part of laboratory research. This is largely due to a significant gap between the computational and experimental sides of the science [1]. Specifically, many computational models (as well as software to simulate and analyze these models) involve complex mathematics, and hence are limited in their utility to those with extensive training in computational methods (modelers). In order to couple computational models more closely with experimental studies, software tools to build and simulate models in a non-mathematical fashion will be required to bridge this gap. [2]–[6]. While some tools (e.g., GINSim [7] or Genetic Network Analyzer [8]) allow users to easily “draw” logical models, for systems with more complex interactions, users are required to manually define the models' underlying mathematics.

In this paper, we present a new tool, Bio-Logic Builder, which allows those without technical knowledge in modeling to build and modify complex computational, qualitative models without the need to write or edit any mathematical equations. Becuase models created in Bio-Logic Builder utilize a commonly used logical (Boolean) mathematical framework (e.g., [9]–[12]), no kinetic parameters (which are generally unavailable or difficult to obtain) are necessary to specify individual biological/biochemical interactions. Specifically, interactions defined using the Bio-Logic builder are described by Boolean expressions that users build by using qualitative descriptives (or “bio-logic” components) generally used by laboratory scientists to explain the interaction from experimental studies (e.g., activators, inhibitors, co-factors, etc.).

The presented Bio-Logic Builder was successfully tested on one of the largest computational models of signal transduction [13] as well a model of ErbB-regulated cell cycle created by another group [14]. Furthermore, we used this tool to construct a budding yeast cell cycle [15], [16], and the largest dynamical model of a regulatory network governing influenza A infection and the virus' replication cycle as part of our most recent research. We found that Bio-Logic Builder was able to handle the regulatory mechanism of all biological species in the models, regardless of the complexity of the mechanism. In the results section, a discussion of the algorithm in more detail, as well as its application to a biological example is provided. Specifically, we will demonstrate how Bio-Logic Builder is used to build a very intricate regulatory mechanism of the Rac protein, which involves 14 upstream regulators. Bio-Logic Builder is part of a web-based modeling suite, The Cell Collective (http://www.thecellcollective.org; [17]) which enables models created using this tool to be also simulated and/or downloaded and used by other modeling tools.

## Results

### Case study: The regulatory mechanism of Rac

Biological interactions defined using the Bio-Logic builder are described by Boolean expressions that users build by using qualitative descriptives (or “bio-logic” components) generally used by laboratory scientists to explain the interaction from experimental studies. Leveraging the qualitative nature in which many biochemical interactions are discovered, Bio-Logic Builder provides users with building blocks of two types. First, users can define modules corresponding to positive and/or negative regulators that are involved in a given biological interaction (e.g., kinase X phosphorylates and activates protein Y, as is the case in studies of biochemical signal transduction). Because only few biological interactions can be represented as simple positive and/or negative regulators, users can specify a second type of bio-logic modules. These modules – “conditions” and “subconditions” – allow users to describe regulatory mechanism in which the effects of one or more positive and/or negative regulators depend on an additional regulators step (e.g., localization, priming, co-factors etc.), and hence the activation state or presence (or absence) of an additional regulator (or group of regulators). As a result, the users can define complex positive and negative regulatory modules much in the same way biological data and knowledge are discovered in the laboratory. To demonstrate how Bio-Logic Builder is used to build biological regulatory mechanisms, in this section is presented a case study which centers around the construction of a relatively complex regulatory system of the signaling protein Rac. Note that a simpler example of how the tool can be used can be viewed in a tutorial video at http://www.thecellcollective.org.

Rac is an important player in the regulation of many cellular processes such as cell migration, cytoskeletal reorganization, DNA synthesis, etc. Rac belongs to the Rho family of small guanosine triphosphatases (GTPases), a subgroup of the Ras superfamily. Rac becomes activated when bound to GTP, a process mediated by guanine nucleotide exchange factors (GEFs). The hydrolysis of GTP to GDP results in the inactive state of Rac. This conversion occurs via Rac's intrinsic GTPase activity and is further accelerated by GTPase-activating proteins (GAPs). However, in addition to GAPs and GEFs, Rac's activity also depends on its proper localization as well as the activity state of components of other signaling pathways. A summary of the intricacies involved in the (de-)activation mechanism of Rac as reported in the biochemical literature so far follows. (Note that the following regulatory mechanism of Rac reflects the optimized mechanism published as part of a validated large-scale model of signal transduction in a generic fibroblast cell [13].)

**Figure 1:**
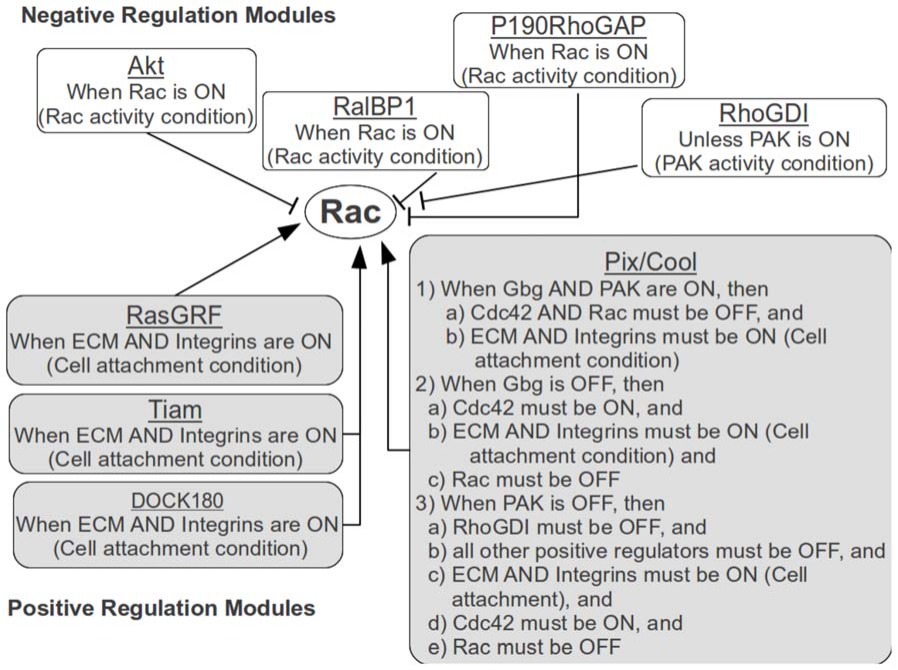
Graphical representation of Rac regulatory mechanism.

In the aforementioned fibroblast model, Rac is defined as ON when it is GTP-bound and localized in the plasma membrane. (See Figure 1 for a graphical summary of the mechanism and the involved species.) RalBP1 [18], [19] and p190RhoGAP [20], [21] are GAPs, and hence negative regulators of Rac when GTP-bound (i.e., active). RhoGDI is also a negative regulator of Rac [20], [22]–[24] because it sequesters GDP/GTP bound Rac. PAK is be able to break up Rac-RhoGDI complex and stop the negative regulation of Rac by RhoGDI [25]. While Akt appears to also be a negative regulator of Rac [26], based on the context of the overall network, it was made not dominant over any of the following positive regulators (and hence does not effect the activity state of Rac). The activation of Rac is mediated by RasGRF [27], [28], Tiam [24], [28], [29], Pix/Cool [30], [31], or DOCK180 [32]–[34]. The effects of these activators is dependent on cell attachment which is represented in the model by the activity of ECM and integrins. However, despite the fact that many of the details haven't been fully discovered, the activation mechanism of Rac by Pix/Cool appears to be relatively complex. In addition to the requirement of cell attachment, there are three different scenarios under which Pix/Cool modulates the activity of Rac. First, when the G protein subunits 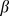 and 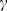 (represented by a single species G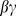) [35] AND PAK are ‘ON ’ Pix/Cool only activates Rac if both Cdc42 AND Rac are ‘Off’. Second, when G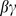 is inactive, Pix/Cool activates Rac only if Rac was previously inactive. In addition, this step also requires the activity of Cdc42. Finally, when PAK is inactive, Pix/Cool activates Rac only when Cdc42 is active, Rac was previously inactive, and RhoGDI, as well all other positive regulators are also inactive [35]–[38]. (Note that due to missing information and/or inconsistencies in biological data, some of the logic of the mechanism might have been adjusted in the context of the whole model.)

As one can see, the regulatory mechanism of Rac is intricate and involves a large number of upstream regulators. Specifically, the activation states of 13 upstream regulators, in addition to the activation state of Rac in the previous time point have to be considered, resulting in 14 regulating inputs of Rac. Thus the truth table representation of the function would require the scientist to manually fill out 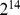 (or 16,384) lines of the species' corresponding table. The complexity of the regulatory mechanism is also matched by the underlying Boolean expression representing the function:
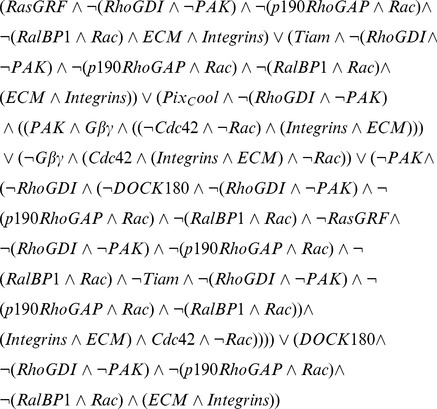
(1)Manual creation of the logical expression for a regulatory mechanism of this size and complexity would be difficult and error prone. Using Bio-Logic Builder, users can capture the complex activation mechanism of Rac in non-mathematical fashion by using the published qualitative information (as described above) and building the regulatory mechanism in a modular fashion as detailed below.

The Bio-Logic Builder tool is part of The Cell Collective modeling suite [17], which can be freely accessed via a web browser by visiting http://www.thecellcollective.org. From the Models page, users can either create a new model, or access any of the existing (e.g., Published) models. New species can be added or existing species modified under the Model Bio-Logic page. The Rac species can be found under the Model Bio-Logic of the “Fibroblast” model (under Published models). Clicking the green “gear wheel” icon next to Rac in the species table takes the user to the Bio-Logic Builder tool where the regulatory mechanism of the (Rac) species can be defined/modified. The first screen allows the user to start building/modifying the regulatory mechanism of Rac by specifying either the positive or negative regulation modules. In this study case, we will start with the negative regulation modules in the Negative Regulation Center. (Note that the order in which the user starts does not result in a different output. Also note that the species in the Published model are read-only for curation purposes; to be able to modify the regulatory mechanisms of the model's species, a “private” copy of the model can be made from the Models page by clicking the “Copy to My Models” icon.)

#### Negative regulation center

**Figure 2:**
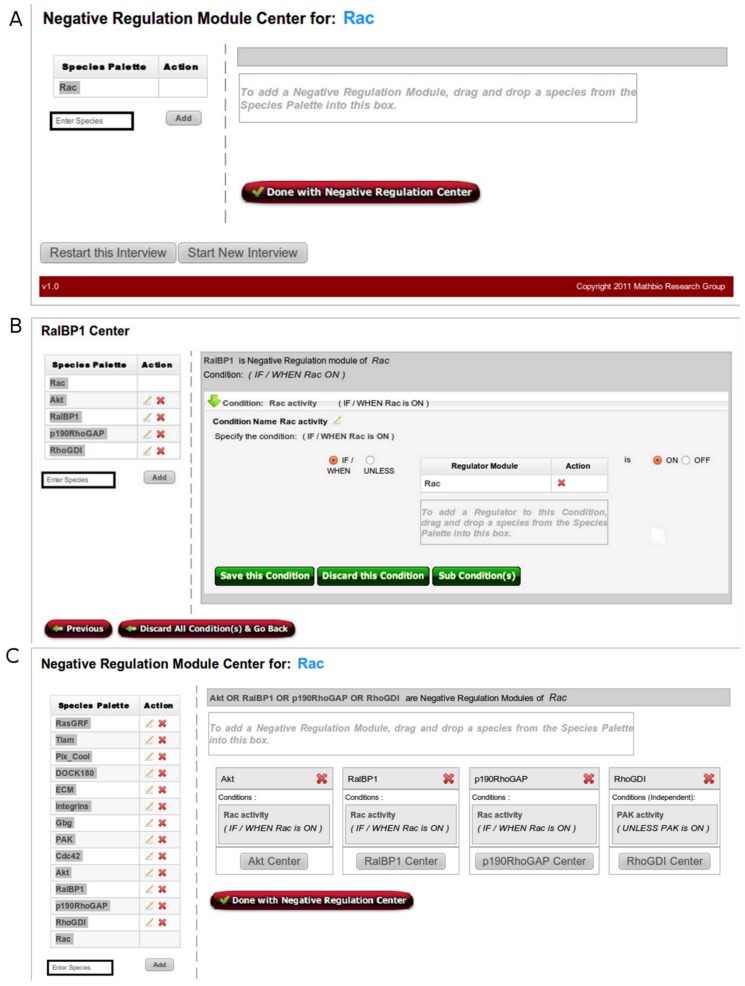
Negative regulation of Rac. A) Main page of the Negative Regulation Center. B) RalBP1 condition page. In order to define the condition RalBP1 is a negative regulator of Rac only when Rac is on, the user first selects the IF/THEN clause. In order to specify Rac as the conditioned species, the user can drag it from the Species Palette into the indicated gray box in the main part of the screen. Finally, the conditioned state of Rac (“is ON”) needs to be selected. In addition, as indicated by the green buttons, the user can subsequently i) save the condition and either return to the Negative Regulation Center page or add another condition for RalBP1, ii) discard this condition, or iii) add one or more sub-conditions that will be attached to the specified condition of RalBP1. The red Previous button takes the user to the previous screen, whereas the Discard All Conditions & Go Back button removes all conditions and returns the user to the Negative Regulation Center. C) Negative Regulation Center of Rac with all modules fully defined.

As the name suggests, Negative Regulation Center is where users designate upstream regulation modules that have a negative (i.e., inhibitory) effect on the species of interest (Rac in this example). From the regulatory mechanism described above, the negative regulators of Rac include Akt, RalBP1, p190RhoGAP, and RhoGDI. As shown in Figure 2A, the left panel of the page displays the “Species Palette” which is responsible for the management of all upstream regulators of Rac. The Species Palette is available to the user throughout the entire building process so that new species can be added/edited as needed. By default, the species for which the regulatory mechanism is being built (i.e., Rac) is automatically added to the palette. Before the user can designate species as negative regulators, they first need to be added to the species palette. Specifying a species as a negative regulator is as simple as drag-and-dropping a species from the palette into the box in the main window (Figure 2).

Once Akt, RalBP1, p190RhoGAP, and RhoGDI are designated as negative regulation modules, conditions can be specified. As discussed in the previous sections, conditions allow biologists to specify regulatory scenarios under which a particular upstream regulator is dependent on the activity state of another species (e.g., a co-factor). In our Rac example, RalBP1 and p190RhoGAP are responsible for removing GTP from a GTP-bound (i.e., active) Rac and replacing it with GDP, hence inactivating Rac. Therefore, the effects of these negative regulators are dependent on the activation state of Rac itself which can be represented as a condition for the two upstream regulators. Based on the context of the whole network in [13], the effect of Akt as a negative regulator is also dependent on the previous activity level of Rac. In the case of Rho-GDI, PAK can break up the Rac-RhoGDI complex, hence a condition also needs to be specified for the RhoGDI module (Figure 2B). Conditions for each of the negative regulation modules are defined under their respective “Centers” as discussed below.

The conditions page is accessed from the Negative (Positive) Regulation Center page by clicking on the Center of the negative (positive) regulation module for which conditions need to be added/modified. For example, the conditions page for RalBP1 can be accessed under the “RalBP1 Center”. Users can define conditions as IF/WHEN and UNLESS statements to define scenarios when the effects of the regulator for which a condition is being specified depend on the activity state of another biological species. As mentioned above, the condition associated with Akt, RalBP1, and p190RhoGAP is that these species are negative regulators IF/WHEN Rac is ON. In addition, for users' convenience, each condition can be annotated to reflect its biological meaning and context. In this case, the condition was named “Rac activity”, but any annotation can be used. In the case of RhoGDI, its effect on the activity of Rac depends on the presence/absence of PAK; specifically, RhoGDI acts as a negative regulator UNLESS PAK is ON. Note that any number of conditions (and subconditions) can be associated with any regulator, allowing for the definition of the most complex regulatory mechanisms. (An example of multi-condition scenario is presented in the next section.) See Figure 2C for the final Negative Regulation Center page which summarizes the complete negative regulation modules of Rac. The “Done with Negative Regulation Center” returns the user to the Center home page where the Positive Regulation Center can be selected.

#### Positive regulation center

Positive regulation modules of the species of interest (e.g., Rac) are specified in the Positive Regulation Center. As discussed at the beginning of the Case Study section, the activating species of Rac include RasGRF, Tiam, Pix/Cool, and DOCK180. Once these species have been added to the Species Palette, they can be defined as positive regulation modules in a similar fashion as was done with the negative regulation modules, and was demonstrated in the Negative Regulation Center section. As the regulatory mechanism suggests, all positive regulators are dependent on cell attachment, and hence the activity of ECM and Integrins. Therefore the positive regulation modules RasGRF, Tiam, and DOCK180 the condition (named Cell attachment) “IF/WHEN ECM AND Integrins are ON”. However, the conditions associated with the positive module Pix/Cool are more complicated. As discussed at the beginning of this case study, there are three nontrivial scenarios describing the role of Pix/Cool in the regulation of Rac activity. To capture this complex regulatory mechanism, both condition and subcondition bio-logic gates are necessary. Subconditions can be specified after clicking the “Subconditions” button on the condition page of the regulation module center.

**Figure 3:**
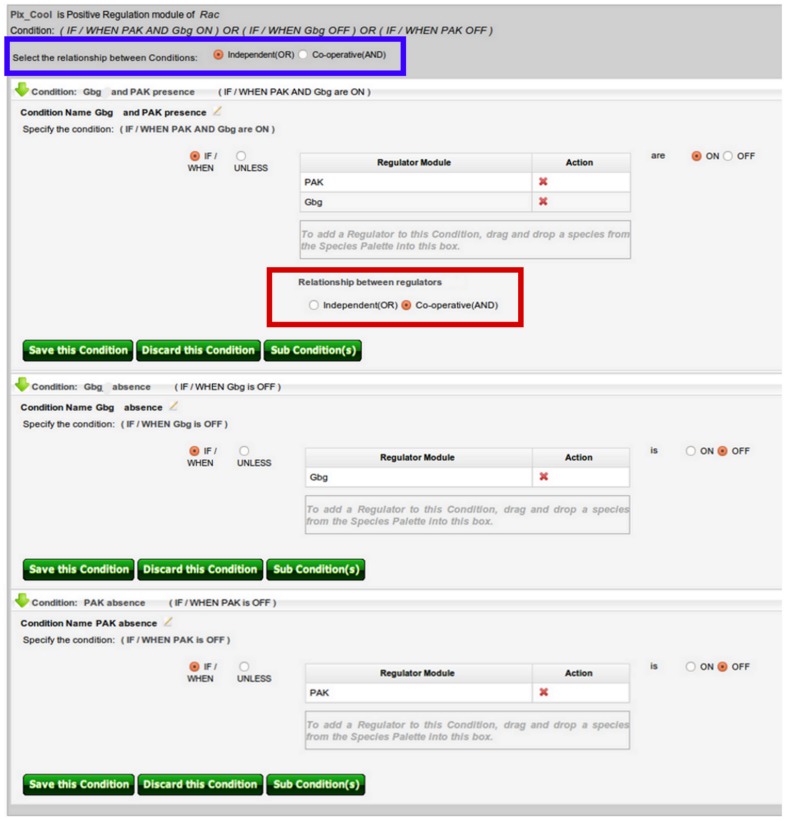
Pix/Cool conditions. As discussed in the main text, the regulation of Rac by Pix/Cool is associated with three different scenarios described by three conditions (and subconditions which are not displayed). When multiple conditioned species are added as part of a condition (as is the case with the first condition of the Pix/Cool module, where the activation states of both PAK and G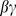 determine the effects of Pix/Cool on Rac), the user first drags the species of interest into the condition box. Subsequently the user is prompted to select the relationship between the species (boxed in red). The available relationships include “Independent” and “Co-operative”, corresponding to the OR and AND Boolean operators, respectively. The Co-operative relationship is selected for the first condition of the Pix/Cool module. Note that any number of conditioned species can be added as part of a condition, and the selected relationship applies to all conditioned species. Similarly when multiple conditions are specified for a regulation module (as is the case with the Pix/Cool module), the user needs to also specify either an Independent or Co-operative relationship for the conditions (highlighted in blue). In the case of Pix/Cool, there are three independent conditions.

**Figure 4:**
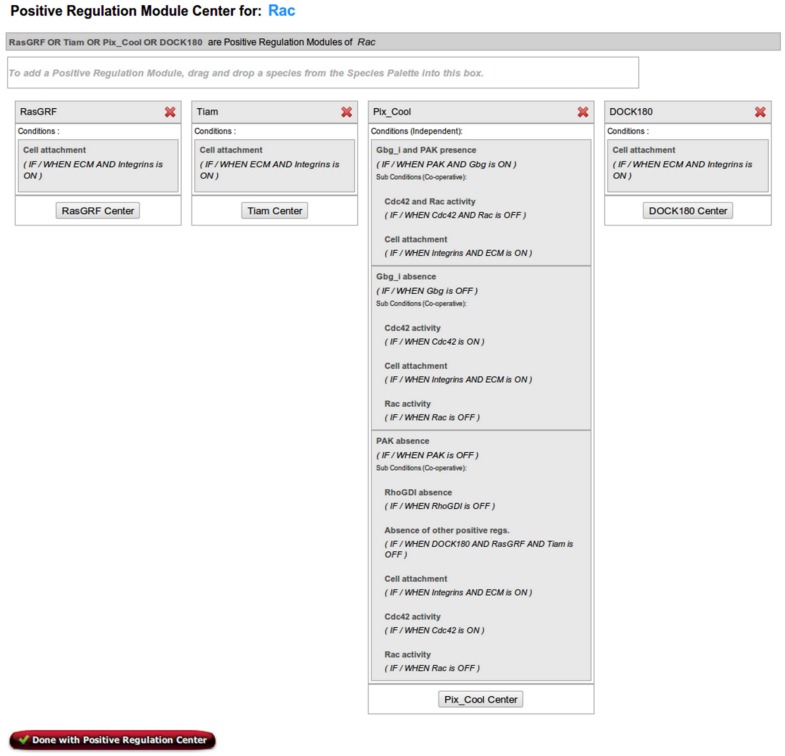
Positive Regulation Center of Rac with all regulation modules fully defined.

The three scenarios differ based on the presence and absence of G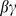 and PAK. First, when G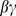 AND PAK are ‘ON’ Pix/Cool only activates Rac if both Cdc42 AND Rac are ‘Off’.In Bio-Logic Builder, the first scenario is represented as a condition “IF/WHEN PAK AND G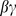 are ON”, followed by subcondition defining the requirement for the absence of Cdc42 and Rac as: “IF/WHEN Cdc42 AND Rac are OFF”. Second, when G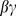 is inactive/absent, Pix/Cool activates Rac only if Rac was previously inactive, and in the presence of Cdc42. This scenario can be represented as a condition “IF/WHEN G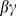 is OFF” which has a subcondition defined as “IF/WHEN Cdc42 is ON”. The third scenario – where contains Pix/Cool activates Rac when PAK is inactive only if Cdc42 is ON, Rac was previously inactive, and RhoGDI and all other positive regulators are OFF – is also defined as a combination of a condition with subconditions. As done in a similar fashion above, to indicate the dependence of Pix/Cool on the absence of PAK, a condition of “IF/WHEN PAK is OFF” is defined. To add the dependence on Cdc42, RhoGDI, DOCK180, RasGRF, Tiam and Rac's previous activation state, the following subconditions are defined in a co-operative manner: “IF/WHEN Cdc42 is ON” (for the dependence on Cdc42 activity), “IF/WHEN RhoGDI is OFF” (for the dependence on the absence of RhoGDI), “IF/WHEN DOCK180 AND RasGRF AND Tiam are OFF”, and “IF/WHEN Rac is OFF” (for the dependence on Rac's previous activation state). In addition, because the activation of Rac by Pix/Cool is also dependent on cell attachment (similar to the other positive regulators), all three conditions have the Cell attachment subcondition (specified above) associated with them. Screen shots in Figure 3 and Figure 4 show the Pix/Cool condition page and the summary page of all positive regulation modules, respectively.

Once all negative and positive regulation modules are defined, the user is led to the next screen, the Dominance Page. On this page, users can define the “strength” of each negative module in terms of how dominant it is over the individual positive regulation modules. A negative module dominant over all positive regulators (pre-selected by default) has the largest (negative) effect on the state of the species of interest, whereas a negative module dominant over none of the positive modules will have no effect on the activity of the species.

**Figure 5:**
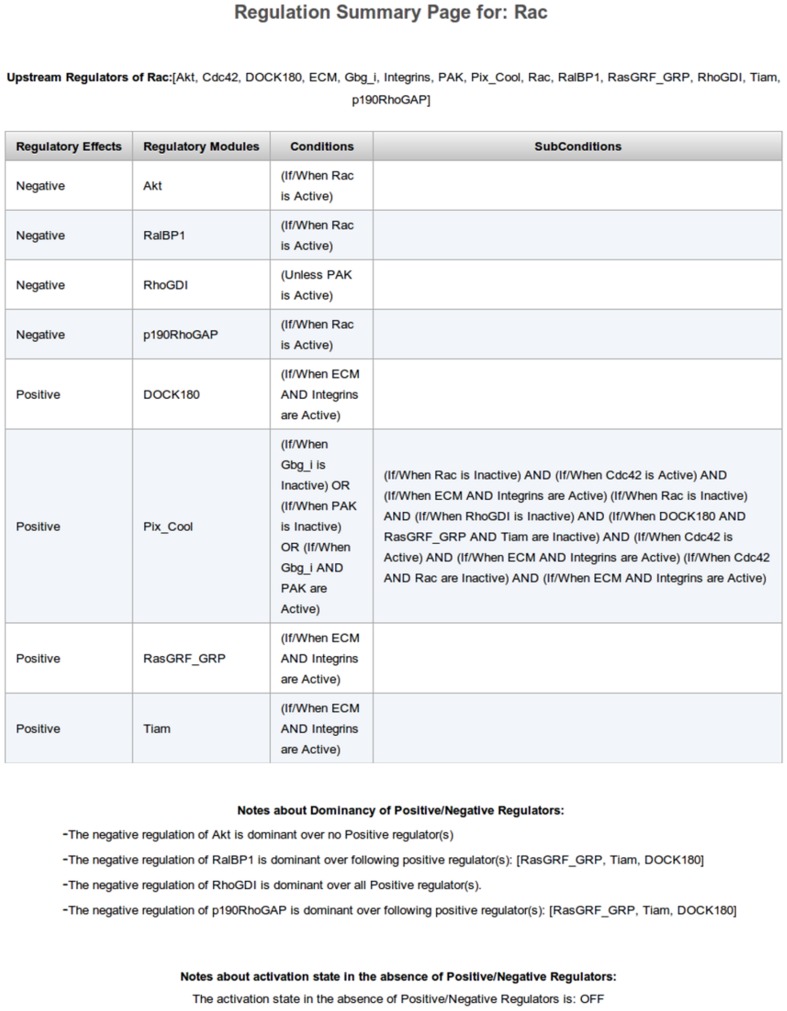
A screen shot of the Summary Page of the Rac regulatory mechanism.

Once the strength of the negative regulation modules is selected, the user needs to specify the final component of the regulatory mechanism building process – the state (active/inactive) of the species in the case where none of the positive nor negative modules are active or present in the cell (model). Upon the last page and component of the Bio-Logic Builder tool, the user can navigate to the Summary Page (Figure 5). This page displays all regulatory modules involved in the regulatory mechanism of Rac. The program builds the mathematical function based on the regulatory modules specified by the user, and constructs the appropriate truth table in the background. The generated truth table can then be plugged into a larger model and simulated/analyzed by one of the software tools mentioned in the Introduction section. Specifically, ChemChains, as described in [39], directly supports logical models represented as truth tables and can be used to easily simulate models created with Bio-Logic Builder. The truth table and logical expression for individual species can be downloaded from the model species page, or a set of all Boolean expressions and truth tables, as well as the SBML file for the entire model can be downloaded from the main Models page in The Cell Collective.

#### Defining the “head regulator” of a positive/negative regulation module

In Bio-Logic Builder, the head regulators represent the main positive/negative regulation modules, within which conditions and subconditions are subsequently added. In the Rac case study presented in this section, the head regulators of the negative regulation modules included Akt, RalBP1, p190RhoGAP, and RhoGDI, whereas the head regulators of the positive regulation modules constituted RasGRF, Tiam, Pix/Cool, and DOCK180 (Figure 1). All of these head regulators had one or more conditions (and subconditions in the case of Pix/Cool for example), and hence forming the corresponding regulation modules. However, what if it is not clear as to which species should be considered the head regulator and which species should be the condition building block of the regulation module? How does one decide which way it be depicted? Does it matter (in terms of the mathematical representation) which way the module is represented?

While for many biological interactions it is clear (based on the available published data) which of the species is considered the “head regulator”, there are many instances in which regulatory mechanisms can be ambiguous and hence become confusing to the user of Bio-Logic Builder. These few regulatory mechanisms can even be relatively simple in terms of the number of species involved in the interaction. For example, consider a hypothetical biochemical signaling protein X with two phosphorylation sites in its regulatory region. Let's assume that, in order to be fully activated, both of the phosphorylation sites of X need to be phosphorylated, one by kinase Y and the other one by kinase Z. From this described situation, one could consider both kinases as “equal” rather than as a “head regulator”/“condition” relationship. However, based on the Rac case study and the previous discussions of the Bio-Logic Builder algorithm, one of the kinases (Y or Z) has to be considered the head regulator, whereas the other one is represented as a condition (IF/WHEN ON) as part of the regulation module. Which way this scenario should be defined, however, is not clear in this example. Nonetheless, it is important to note that when a number of regulation species appear to be conceptually equal, Bio-Logic Builder requires one of these species be selected as the head regulator whereas the others be considered as a (sub)condition(s). Fortunately, because of the mathematical relationship between the head regulators and the conditions, the mathematical representation of the interaction will be the same in both cases (as detailed in Supporting Information S1). If such a scenario arises, the user will need to use their discretion and decide how to represent the regulatory mechanism.

### Algorithm verification

The *scalability*, *uniqueness*, *predictability*, as well as the *correctness* of the algorithm underlying Bio-Logic Builder were tested. The algorithm scalability was addressed by showing (mathematically) that any Boolean expression for an 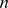-input node can be created using Bio-Logic Builder (see Supporting Information S1). For uniqueness and predictability, we show that given unique combination of user inputs, Bio-Logic Builder generates a predictable, however, not globally unique result. In other words, users can obtain the same truth table with 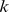 different sets of regulation modules (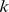 has not been enumerated). On the other hand, it is not possible for a set of user-defined regulation modules to generate more than one unique Boolean function. Hence, Bio-Logic Builder is unique in an unidirectional fashion. This 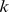:1 relationship between sets of user inputs and the unique Boolean functions provides users with flexibility in the conceptual interpretation of a given regulatory mechanism. Detailed discussions of scalability, uniqueness, and predictability can be found in the *Scalability of the Bio-Logic Builder Algorithm* and *Predictability, Uniqueness and Correctness of the results of the Bio-Logic Builder Algorithm* sections in Supporting Information S1, the results of these analyses of the algorithm are summarized in this section. The correctness of the algorithm is also addressed in Supporting Information S1. Therein we detail how Bio-Logic Builder generates the correct Boolean function of a biochemical species using the defined set of positive/negative regulation modules.

### Usability and intuitiveness of the graphical user interface

The usability and intuitiveness of Bio-Logic Builder was tested by creating some of the most complex regulatory mechanisms included in one of the largest models of signal transduction (135 molecular species with hundreds of biochemical interactions, [13]), as well as other models such as yeast cell cycle [15], [16], ErbB-stimulated G1/S cell cycle transition [14], and Influenza A replication cycle (manuscript in preparation, however, the model is available in The Cell Collective [17]).

### Model simulations

Because models constructed in the presented tool are described using standard Boolean formalisms, they can be simulated by a large number of software tools. In addition to the previously mentioned Cell Collective (whose simulation engine is based on ChemChains [39]), additional examples of tools able to simulate Boolean models include BooleanNet [40], BoolNet [41], GINSim [7], or Genetic Network Analyzer [8].

## Conclusions

The lack of simple-to-use tools for creating/editing and simulating computational models plays a significant role in the gap that exists between the computational and experimental sides of biomedical research [6]. With Bio-Logic Builder, we were able to capture the qualitative nature of computational models and translate it into a math-friendly and relatively intuitive user interface that allows users to build complex biological interaction without the need to enter or edit any mathematical equations. This was accomplished by generalizing the relationships between biological regulatory components to qualitative relationships, or bio-logic gates. To the users, these bio-logic gates appear as black-boxes in which the mathematical functions are generated in the background based on the gates' properties (i.e., conditions and subconditions). Using purely qualitative information directly generated in the laboratory and published in the literature, we demonstrated how Bio-Logic Builder is used to build the 14-component regulatory mechanism of the Rac protein without the need for a direct interaction with mathematical equations. To test the scalability of the software we show (mathematically) that the algorithm can be used to build any mathematical representation of a biological process. Furthermore, to verify the user-intuitiveness of the tool, we used Bio-Logic Builder to create some of the most complex signaling regulatory mechanisms of an existing models [13], [14], [16] All of these models are available to the scientific community in The Cell Collective (http://wwww.thecellcollective.org; [17]). A current limitation of the software includes the inability to import models created by others. This is largely due to the fact that current exchange standards such as SBML lack support for qualitative models. However, a new extension of SBML, compatible with these models, is already under development (http://sbml.org/Community/Wiki/SBML_Level_3_Proposals/Qualitative_Models). Once its development is completed, a feature to import models will be also.

## Methods

### Implementation

Bio-Logic Builder is a server-based tool implemented in Java and powered by MySQL database. The user interface was implemented primarily using JavaServer Faces (http://www.javaserverfaces.org) and Primefaces (http://www.primefaces.org).

### Boolean models

Boolean modeling is a (kinetic) parameter-free modeling approach based on qualitative data (e.g., kinase *X* phosphorylates and activates protein *Y*). Boolean models consist of i) components (or nodes) that correspond to biochemical species (such as proteins, genes, etc.) and ii) directed edges representing the interactions between the components (e.g., protein-protein interactions). Each node can assume one of two states - ON/active or OFF/inactive (or 1 and 0, respectively).

**Figure 6:**
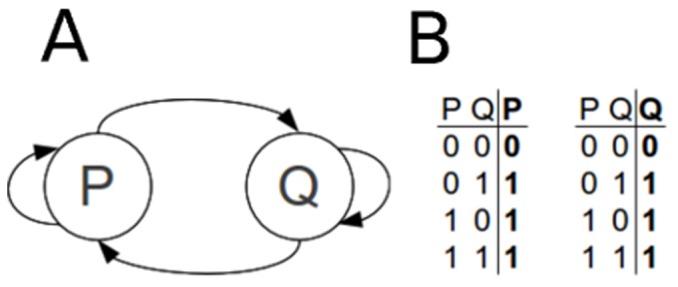
A sample two-node toy model. A) Static diagram representing the relationship between the nodes. B) Truth table representations of the Boolean functions for nodes P and Q.

Which state a node will assume at any given time is determined by the node's Boolean function. Boolean functions can be represented in various ways such as truth tables or Boolean expressions. As an example, consider a hypothetical simple two-node model in Figure 6A. In this particular model, node 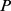 activates node 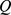 and vice versa. In addition, both nodes can self-activate.

The truth tables depicting regulatory mechanisms of nodes 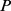 and 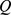 are illustrated in Figure 6B. The left-hand side of the tables contains all theoretically possible combinations of the ON/OFF states (of which there are 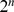) of the input nodes, whereas the right-most column of the tables corresponds to the Boolean value of the node (referred to as “output node”) for the particular state combination of the input nodes. As can be seen from the truth table representation, the functions of both nodes 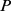 and 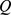 correspond to a simple OR function, which can also be symbolically represented as a Boolean expression as:
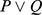



### User input and algorithm structure

#### Main regulators

At the most basic level of a biological regulatory mechanism, users can define positive regulation and negative regulation modules (activator and inhibitors, respectively). Users also define the dominance of individual negative regulators over positive regulators (if applicable). Finally, users specify the state of the biological entity in the absence (i.e., inactivity) of all positive and negative regulators. These regulatory definitions are subsequently used by the software to create the Boolean function representing a regulatory mechanism of a given species as follows.

**Figure 7:**
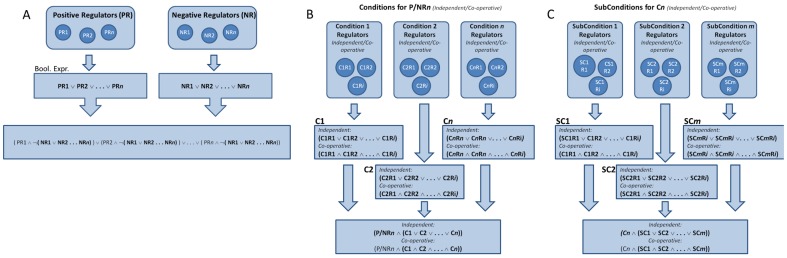
Bio-Logic Builder algorithm for construction of Boolean expressions.

All main positive/negative regulators are defined as independent modulators of the given species. Hence the Boolean expression is constructed by concatenating all positive regulators using the Boolean OR operator (Figure 7A). As also illustrated in the figure, negative regulators defined in the Bio-Logic builder are then appended (AND‘ed) in a negated form to the positive regulators. The set of negative regulators that is appended to each individual positive regulator is determined by the dominance of the corresponding negative regulator selected by the user. For example, if NR1 and NR2 are specified to be dominant over PR1, the corresponding part of the Boolean expression will be the following:
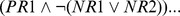
If NR3 is specified as the only negative regulator dominant over PR2, the Boolean expression will be constructed as:
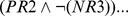



#### Conditions and subconditions

Users may define complex regulatory mechanisms using conditions and subconditions that are applied to a (positive/negative) regulation module. Each positive or negative regulator can have *n* conditions. Each condition is constructed as a Boolean expression substring which is subsequently appended (AND‘ed) to the positive or negative regulatory element (Figure 7B). Furthermore, each condition can have *m* subconditions. Similar to the conditions, each subcondition is a Boolean expression substring that is appended (AND‘ed) to the corresponding conditio (Figure 7C).

#### Co-operative versus independent relationships

When defining a condition or subcondition, multiple regulators may be specified. In this case, the user must specify the relationship between the regulators. This may be co-operative or independent. As illustrated in Panels B and C in Figure 7, in a co-operative relationship, the elements are combined with the AND operator, whereas in an independent relationship, the elements are combined with the OR operator.

As mentioned in the *Conditions and subconditions* section above, multiple (sub)conditions can be defined. Similar to having multiple components for each (sub)condition, the user must specify the co-operative or independent relationship for the (sub)conditions that are subsequently combined to the corresponding Boolean expression (Figure 7B and C).

#### IF/WHEN and UNLESS statements of conditions and subconditions

Each (sub)condition is defined by combining the “IF/WHEN” or “UNLESS” statements with a list of regulators that are specified as either Active or Inactive. In terms of a Boolean expression, the specifying IF/WHEN Inactive or UNLESS Active for a given (set of) regulators corresponds to the Boolean NOT operator. (As mentioned above, multiple (sub)condition regulators are defined, the user must specify whether the regulators are co-operative or independent.

For example, the condition “IF/WHEN A, B, C ARE OFF” (A, B, and C are co-operative regulators) corresponds the following Boolean expression:
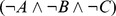
Also, “UNLESS A B C IS ON” (A, B, and C are independent regulators) is interpreted as:
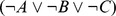



### Software output

Once a user defines the regulatory mechanism of a biological species of interest, Bio-Logic Builder generates the corresponding Boolean function in the form of a Boolean expression as well as a truth table which can be saved in text and tab-delimited (.csv) files, respectively. Because both formats are standard representations of Boolean functions, they can be subsequently read and evaluated by other simulation software tools. Furthermore, the regulatory mechanisms defined in Bio-Logic Builder can be also exported in the Systems Biology Markup Language format (SBML [42]; based on the most recent version of the L3 extension for qualitative models [43]).

### Availability and requirements

Bio-Logic Builder is freely available as part of The Cell Collective modeling platform (http://www.thecellcollective.org; [17]). The software has been optimized for the Firefox and Chromium web browsers, and also works in the Opera and Internet Explorer web browsers.

## Supporting Information

Supporting Information S1 **Detailed description and analysis of the Bio-Logic Builder algorithm.**
(PDF)
