# Supplementary material for: Bio-Logic Builder: A Non-Technical Tool for Building Dynamical, Qualitative Models
Source: PLoS One. 2012 Oct 17;7(10):e46417. doi: 10.1371/journal.pone.0046417 (PMC3474764; doi:10.1371/journal.pone.0046417)
Supplement: Supporting Information S1 — Detailed description and analysis of the Bio-Logic Builder algorithm. (PDF) [file pone.0046417.s001.pdf]

Bio-Logic Builder: Supplementary Information

Tomáš Helikar[1,*], Bryan Kowal[2], Alex Madrahimov[1,3], Manish Shrestha[1], Jay Pedersen[2], Kahani Limbu[2], Ishwor Thapa[2], Thaine Rowley[1,3], Rahul Satalkar[2], Naomi Kochi[3], John Konvalina[1], Jim A. Rogers[1,4]

1 Department of Mathematics, University of Nebraska at Omaha, Omaha, NE, USA

2 College of Information Science and Technology, University of Nebraska at Omaha, Omaha, NE, USA

3 Department of Biology, University of Nebraska at Omaha, Omaha, NE, USA

4 Department of Genetics, Cell Biology and Anatomy, University of Nebraska Medical Center, Omaha, NE, USA

∗ **E-mail: thelikar@unomaha.edu**

Creating a Boolean State Function with Bio-Logic Builder

The Bio-Logic Builder web application is used to define the Boolean state function associated with a biological entity for use in a Boolean Network. It allows the user to define positive and negative regulation modules which describe the function of the biological entity using Boolean logic. Regulation modules with arbitrarily complex Boolean logic may be defined.

This document presents a detailed analysis of the Bio-Logic Builder algorithm. It shows the algorithm to be scalable, predictable, and correct. Scalable refers to the ability to model any boolean function, regardless of the number of boolean variables involved. Correctness refers to the behavior of the generated Boolean function matching the behavior descriptions given to users of the application.

Some basic background in Boolean logic is assumed, such as understanding the concepts of Truth Tables, the Boolean functions AND, OR and NOT, and DeMorgan's laws for distributing the NOT operator. The typical Boolean Algebra convention of using the operators $+$ and $*$ to represent the OR and AND operators [1], and 0 and 1 to represent $TRUE$ and $FALSE$ is used. The reader interested in additional technical detail regarding Boolean logic may examine the Reference section [1–4].

[1]Thus, a sum of Boolean terms applies the OR operator to those terms, and a product of Boolean terms applies the AND operator to them.

Variables and Expressions used by the Algorithm

This section defines the variables and expressions used by the Bio-Logic Builder application. They fully define the output Boolean state function of the application. In fact, the $RESULT$ expression is the result of the application.

- n – the number of species in the species palette
- x_1 to x_n – Boolean variables representing the activation state of the n species
- p, q – the count of positive regulators and negative regulators, respectively
- pr_i, nr_j – the ith positive regulator and jth negative regulator, respectively, where i is from 1 to p and j is from 1 to q. Each regulator is a species from the species palette and is in fact x_k, for some k from 1 to n.
- PRC_i, NRC_j – the Boolean expression for the conditions and subconditions applied to the ith positive regulator and jth negative regulator, respectively. Each defaults to TRUE if no conditions are specified.
- $PR_i = (pr_i \text{ AND } PRC_i)$ – the Boolean expression for the ith positive regulator including its conditions and subconditions
- $NR_j = (nr_j \text{ AND } NRC_j)$ – the Boolean expression for the jth negative regulator including its conditions and subconditions
- $PRoverride_i$ is the set of indexes of negative regulators which override PR_i. For example, $PRoverride_2 = \{1, 3\}$ indicates that the negative regulators expressions NR_1 and NR_3 will be applied to PR_2.
- $PRO_i = \begin{cases} TRUE, & PRoverride_i = \{\} \\ \prod_{j \in PRoverride_i} \neg NR_j, & \text{otherwise} \end{cases}$
- $ABSENT = \begin{cases} \text{FALSE}, & \text{user answered “OFF” as the activitity level when ALL regulators are OFF} \\ \prod_{i=1}^{n} \overline{x_i}, & \text{user answered “ON”} \end{cases}$
- $POSNEG\_REGULATORS = \begin{cases} FALSE, & \text{p}= 0 \\ \sum_{i=1}^{p} PR_i \text{ AND } PRO_i, & \text{otherwise} \end{cases}$
- $POS\_REGULATORS = \begin{cases} FALSE, & \text{p}= 0 \\ \sum_{i=1}^{p} PR_i, & \text{otherwise} \end{cases}$
- $RESULT = POSNEG\_REGULATORS$ OR $ABSENT$

The $ABSENT$ expression is also called the $ABSENT$ regulator.

Scalability of the Algorithm

This section describes how to generate any possible Boolean function of n-inputs using Bio-Logic Builder. Definitions from the Variables and Expressions section are used.

A Disjunctive Normal Form (DNF) of a Boolean function is a summation of distinct complete products. A complete product is a product of all the Boolean variable arguments of a Boolean function, where each variable is specified exactly once in either complemented (negated) or uncomplemented form. It can be easily shown that there is a DNF representation of any Boolean function $f \neq 0$.

At most one term in the DNF representation of a Boolean function can have all inputs negated, as that is a distinct complete product. The $ABSENT$ regulator controls whether this term is in the generated Boolean function.

The Boolean function created by the Bio-Logic Builder is exactly the $RESULT$ expression, which is $POSNEG\_REGULATORS$ OR $ABSENT$.

If negative regulators are not specified, then $POSNEG\_REGULATORS$ simplifies to $POS\_REGULATORS$, and the generated Boolean function generated is $POS\_REGULATORS$ OR $ABSENT$.

Procedure for implementing an arbitrary Boolean function

This procedure is written from the perspective of a user of Bio-Logic Builder. It is not used by Bio-Logic builder itself. Given an arbitrary Boolean function, we may craft inputs to Bio-Logic builder which will cause it to create that function as its output.

Let BF be an arbitrary Boolean function.

Only positive regulators and the $ABSENT$ regulator will be used in this procedure. This results in the Boolean function $POS\_REGULATORS$ OR $ABSENT$ being generated.

Use the following steps to determine how to define the positive regulators and the $ABSENT$ regulator. This will result in the generation of a Boolean function equivalent to BF.

Since there are no negative regulators, the positive regulators will be the n species in the species

palette, which we may label x_1 to x_n.

For the trivial case of $BF = 0$, create a single positive regulator x_1 with the condition "IF/WHEN x_1 IS OFF". Set the $ABSENT$ regulator to OFF and enter no other regulators. This generates the Boolean function $x_1\overline{x_1}$ OR $FALSE$, which evaluates to $FALSE$ and matches $BF = 0$.

The rest of the procedure deals with the non-trivial case of $BF \neq 0$.

Let BE be a DNF form Boolean expression for BF. BE exists as $BF \neq 0$. Thus, BE is a non-empty summation of complete product terms.

Let $ALLINPUTSOFF = \prod_{i=1}^{n} \overline{x_i}$. This is the "all inputs negated" term that is controlled by the $ABSENT$ regulator.

For the special case of $BF = ALLINPUTSOFF$, set the $ABSENT$ regulator to ON and define a single positive regulator x_1 with the condition "IF/WHEN x_1 IS OFF". This generates the Boolean function $x_1\overline{x_1}$ OR $ALLINPUTSOFF$ which simplifies to $ALLINPUTSOFF$.

We now consider all other cases, where $BF \neq 0$ and $BF \neq ALLINPUTSOFF$.

Let S be the set of complete products in BE, excluding the possible $ALLINPUTSOFF$ term. S is non-empty as $BE \neq ALLINPUTSOFF$ and $BE \neq 0$ and thus must contain at least one complete product other than $ALLINPUTSOFF$.

Every term in S must have at least one non-negated input, as the only term which every input is negated is the $ALLINPUTSOFF$ term.

We may partition these terms into n partitions based on the lowest-index value of the non-negated variables in each term. The first partition will contains terms with x_1 (non-negated). The second partition contains terms with $\overline{x_1}x_2$ (as x_2 is the lowest-indexed non-negated input in any such term). The third partition contains terms with $\overline{x_1 x_2}x_3$, and so on. The final partition is the nth partition and contains the term $(\prod_{i=1}^{n-1} \overline{x_i})x_n$, if it exists in S.

Since all terms have at least one non-negated input variable, this partitioning includes all terms in S.

There are n partitions, and at least one is non-empty. This construction allows us to associate the ith

partition with variable x_i, which is the lowest-indexed non-negated variable of each term in the partition.

For each non-empty partition i, perform the following in Bio-Logic Builder:

Create positive regulator x_i.

Create conditions and subconditions for regulator x_i which causes its Boolean Expression to equal to the sum of the complete product terms in the partition. This can be done as follows:

- Create one condition for each term, specifying that the conditions are independent.
- For each condition:
  - Specify the non-negated variables in the term as co-operative and use the "WHEN x IS ON" clause. Note that this will always contain the variable x_i.
  - If there are negated variables in the term, create a subcondition which contains them and specifies that they are co-operative and use the "WHEN x IS OFF" clause.

Due to the definition of PR_i, x_i will be ANDed with the sum of these complete product terms. This distributes x_i to each of those terms (by Boolean Algebra definitions). Because each term contains x_i and the fact that (x_i AND x_i) = x_i, the resulting PR_i will have the value of the sum of the complete product terms in the partition.

Since this will be done for each non-empty partition, every complete product term in S will be accounted for and summed and the result of the POS_REGULATORS definition will be the sum of all complete product terms in S.

Note that we have already defined the $ABSENT$ regulator to be either $ALLTERMSOFF$ or $FALSE$ (if the "all inputs negated" term is not present in BF). This accounts for the only possible complete-product term in BF that was not in S.

Therefore the generated Boolean function will be the sum of all complete products in BF. This is exactly the definition of DNF Boolean form for BF. Thus, Bio-Logic Builder will create a Boolean function equivalent to BF.

This completes the procedure definition.

Note that the procedure involves only a linear sequence of steps; there are no recursive steps involved.

Regardless of the number of variables in the Boolean function, the procedure only needs conditions and subconditions to define it. The conditions are used to define the non-negated variables in each term of each defined partition. The subconditions are used to define the negated variables in each term of each partition. Thus, there is no limitation on n, based on the fact that there are only conditions and subconditions in the model.

Conclusion

Bio-Logic Builder is capable of creating any arbitrary Boolean function definition, regardless of its complexity. One way this can be done is by defining the $ABSENT$ and positive regulators as described by the procedure above, and skipping all other regulator definitions.

Predictability, Uniqueness and Correctness of the results of the Algorithm

Uniqueness of the Result

Every unique input to the algorithm results in a predictable, but not globally unique result. That is, multiple inputs to the algorithm can result in the same output being generated.

For example, it can be shown that there are at least two different Bio-Logic Builder inputs which generate Boolean function A OR B for a two-input element with inputs A and B.

Specifically:

1. $ABSENT$=OFF and PR_1=A and PR_2=B
2. $ABSENT$=OFF and PR_1=A and PR_2=B and PR_2 has the condition "WHEN B IS ON"

Predictability of the Result

Any unique input to Bio-Logic Builder results in a unique setting of key variables and expressions used by the algorithm (as defined in the Variables and Expressions section).

Bio-Logic Builder will generate a Boolean function of n variables whose closed form expression is given by $POSNEG\_REGULATORS$ OR $ABSENT$. This is completely predictable and deterministic for any possible setting of input values.

Correctness of the Result

This section is concerned with the correctness of the algorithm. That is, whether a biologist using the Biological Operators (positive/negative regulators and conditions/subconditions) will obtain the correct logical definition of a protein.

It has been shown that the results are predictable for any input, but are they correct from the

standpoint of a biologist using the application? Bio-Logic Builder allows the biologist to define positive regulators, negative regulators and the $ABSENT$ regulator. It will be shown that the Boolean function created will match the definitions given to the biologist for using the application.

In this section, the phrase "regulator is active" includes the conditions and subconditions of that regulator. That is, a positive regulator is only considered active when its Boolean expression which includes its conditions and subconditions evaluates to $TRUE$. That is, the expression PR_i or NR_j is being discussed in this context.

Positive Regulators and Negative Regulators

There are multiple cases to consider:

- no positive or negative regulators defined
- only positive regulators defined
- only negative regulators defined
- positive and negative regulators defined

Note that this section refers to definitions in the Variables and Expressions section.

No Positive or Negative Regulators defined. In this case the $POSNEG\_REGULATORS$ simplify to $FALSE$ and therefore the Boolean function evaluates to FALSE OR ABSENT which simplifies to $ABSENT$. Thus, if no positive or negative regulators have been defined, the function output depends on the $ABSENT$ regulator. This matches the definition given to the biologist.

Only Positive Regulators defined. In this case, there are no negative regulators and the Boolean function evaluates to POS_REGULATORS OR ABSENT. Therefore, if any positive regulators are active, the Boolean function output will be $TRUE$. This is because POS_REGULATORS will evaluate to $TRUE$, resulting in the function output of TRUE OR ABSENT which simplifies to $TRUE$. If all positive regulators are inactive, the function output will depend on the $ABSENT$ regulator as the function will evaluate to FALSE OR ABSENT which simplifies to $ABSENT$. This matches the definition given to the biologist for positive regulators which do not have associated negative regulators.

Only Negative Regulators defined. In this case, there are no positive regulators and $POSNEG\_REGULATORS$ simplifies to $FALSE$ and the Boolean function evaluates to $FALSE$ OR $ABSENT$ which simplifies to $ABSENT$. Thus, if only negative regulators have been defined, the function output depends on the $ABSENT$ regulator. This matches the definition given to the biologist.

Both Positive and Negative Regulators defined. In this case, we have both positive and negative regulators and the negative regulators are "counteracting" the positive regulators.

For example, when negative regulators y and z override positive regulator x, the result is the Boolean expression (PR_x AND NOT NR_y AND NOT NR_z).

In this case, if any positive regulator is active and not counteracted by an active negative regulator, then the Boolean function output will be $TRUE$. This is because $POSNEG\_REGULATORS$ will evaluate to $TRUE$ and the function output will be ($TRUE$ OR $ABSENT$) which simplifies to $TRUE$. If each positive regulator is either inactive or overridden by an active negative regulator, then the function output depends on the $ABSENT$ regulator. This is because the function output will evaluate to ($FALSE$ OR $ABSENT$) which simplifies to $ABSENT$. This matches the definition given to the biologist for positive regulators with associated negative regulators.

The ABSENT Regulator

The Boolean expression for the $ABSENT$ regulator is defined to be $FALSE$ if the target state is specified as OFF, otherwise it is the product of the negations of all inputs ($\overline{x_1 \ldots x_n}$). It is not possible to generate the Boolean term $\overline{x_1 \ldots x_n}$ in any other way.

If the $ABSENT$ regulator is specified as OFF, the definition guarantees that the Boolean function output will be $FALSE$ when all regulators are inactive. This matches the definition given to the biologist.

If the $ABSENT$ regulator is specified as ON, the definition guarantees that the Boolean function output will be $TRUE$ if all regulators are inactive. This matches the definition given to the biologist.

Conclusion

It has been shown that the Boolean function generated by Bio-Logic Builder matches the definitions given to the biologist and can therefore be considered to be correct.

Conclusion

This document presented a detailed analysis of the Bio-Logic Builder algorithm for generating Boolean state functions for biological entities. It showed the algorithm to be scalable, predictable, and correct.

References

1. Gerald E. Williams, Boolean Algebra with Computer Applications, (McGraw-Hill Book Company, 1970)
2. Elliot Mendelson, Boolean Algebra and Switching Circuits, (McGraw-Hill, 1970)
3. Franz E Hohn, Applied Boolean Algebra, 2ed, (The MacMillan Company, New York, 1966)
4. J. Eldon Whitesitt, Boolean Algebra and its Applications, (Addison-Wesley Publishing Company, Reading, Massachusetts, 1961)
